# Developmentally regulated local inhibition and loss of plasticity hinder reinnervation of the skin by injured peripheral sensory axons

**DOI:** 10.1016/j.cub.2009.10.051

**Published:** 2009-12-03

**Authors:** Georgeann S. O’Brien, Seanna M. Martin, Christian Söllner, Gavin J. Wright, Catherina G. Becker, Carlos Portera-Cailliau, Alvaro Sagasti

**Affiliations:** 1 Department of Molecular Cell and Developmental Biology, University of California, Los Angeles, California, 90095, USA; 2 Cell Surface Signalling Laboratory, Wellcome Trust Sanger Institute, Hinxton, Cambridge CB10 1HH, United Kingdom; 3 Centre for Neuroregeneration, School of Biomedical Sciences, University of Edinburgh, Summerhall, Edinburgh EH9 1QH, UK; 4 Departments of Neurology and Neurobiology, David Geffen School of Medicine at UCLA, Los Angeles, California, 90095, USA

## Abstract

The structural plasticity of neurites in the central nervous system (CNS) diminishes dramatically after initial development, but the peripheral nervous system (PNS) retains substantial plasticity into adulthood. Nevertheless, functional reinnervation by injured peripheral sensory neurons is often incomplete [1–6]. To investigate the developmental control of skin reinnervation we imaged the regeneration of trigeminal sensory axon terminals in live zebrafish larvae following laser axotomy. When axons were injured during early stages of outgrowth, regenerating and uninjured axons grew into denervated skin and competed with one another for territory. At later stages, after the establishment of peripheral arbor territories, the ability of uninjured neighbors to sprout diminished severely, and although injured axons reinitiated growth, they were repelled by denervated skin. Regenerating axons were repelled specifically by their former territories, suggesting that local inhibitory factors persist in these regions. Antagonizing the function of several members of the Nogo Receptor (NgR)/RhoA pathway improved the capacity of injured axons to grow into denervated skin. Thus, as in the CNS, impediments to reinnervation in the PNS arise after initial establishment of axon arbor structure.

## Results

### Regenerating sensory axons avoid their former territories in the skin at later developmental stages

The peripheral axons of trigeminal sensory neurons terminate in elaborate arbors within the skin of the head to mediate somatosensation. Although it is well known that the primary axons of peripheral sensory neurons possess substantial regenerative capacity [reviewed in 1], few studies have examined the regenerative potential of unmyelinated axon terminals in the skin. We used zebrafish trigeminal neurons as a model to investigate the developmental regulation of cutaneous axon terminal plasticity and whether deficits in skin reinnervation may contribute to incomplete recovery following injury.

**Figure 1:**
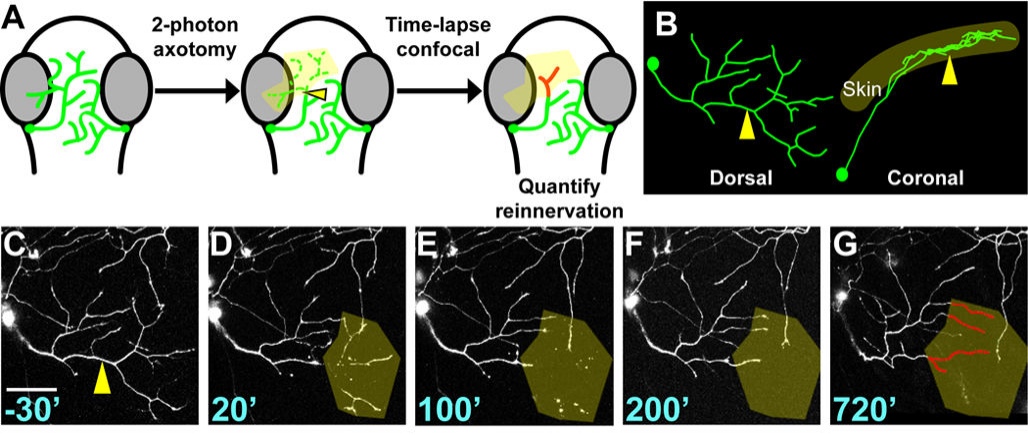
Laser axotomy and imaging of peripheral axon regeneration (A) Experimental design. Dorsal view of zebrafish embryo head; eyes are grey, trigeminal axons are green. Arrowhead indicates site of axotomy in all panels. Axon regeneration was monitored by time lapse for ≥ 12 hours and reinnervation of the denervated territory (yellow shading) by new axon growth (red) was calculated. (B) Dorsal and lateral views of a trigeminal axon reconstructed in 3-D. The axon arborizes within a mostly 2-D plane. (C–G) Time series of confocal image stacks. Same axon as in 1B. Time stamps are in minutes relative to axotomy at 30 hpf. Olive shading highlights the denervated region. Scale bar = 50 μm. See Movie S1.

The skin of larval zebrafish consists of two epithelial layers, between which trigeminal axon terminals reside. These axons repel one another as they arborize to partition epidermal territory, leading to complete, minimally redundant innervation of the head by 36 hours post-fertilization (hpf) [7]. To study the developmental control of skin reinnervation after injury, we severed the peripheral arbors of zebrafish trigeminal neurons expressing green fluorescent protein (GFP) with a femtosecond laser (Figure S1) [8], imaged their regeneration in live embryos for 12 hours, and traced their structure to quantify the success of target reinnervation (Figure 1 and supplemental movies). To specifically study axon regeneration within the skin, all axotomies were directed to second or third order axon branches, which are situated between the two skin layers.

**Figure 2:**
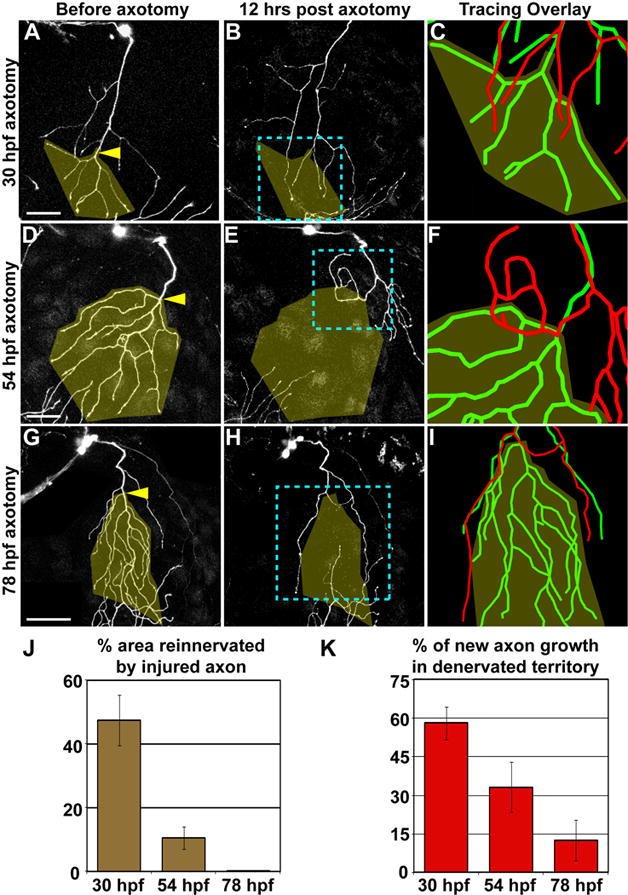
The capacity for reinnervation diminishes during development Axotomy at 30hpf (A–C), 54 hpf (D–F), or 78 hpf (G–I). Left two columns are confocal projections. Scale bars = 50 μm. Yellow arrowheads indicate site of axotomy, and olive overlay marks the territory that was denervated after axotomy. Blue dashed boxes indicate the area represented in rightmost panels (C, F, I), which show 3-D reconstructions of the same axon before axotomy (green) and 12 hours after axotomy (red), aligned at the shared branchpoint most proximal to the axotomy site. (J) Quantification of the average area reinnervated by injured axons, calculated as surface area of regenerated axon in denervated territory/surface area of denervated territory. (K) Quantification of average new axon growth that entered the denervated territory, calculated as length of new growth in denervated region/total length of new growth. Error bars ± S.E.M. See Table S1 and Movies S2–S4.

Axon regeneration was analyzed at three stages: 30 hpf, when trigeminal peripheral axons are still actively arborizing; 54 hpf, ~18 hours after arborization is complete; and 78 hpf. In all cases, the distal portions of severed axons degenerated and were cleared within three hours, leaving a denervated region of skin. Regenerating axons always partially reinnervated their former territories after axotomy at 30 hpf, but their capacity for reinnervation was significantly diminished after axotomy at 54 hpf, and virtually nonexistent at 78 hpf. The portion of former territory reinnervated decreased from 47.5 ± 7.9 % at 30 hpf to 0.3 ± 0.2 % at 78 hpf (p<0.0001); the percent of new growth that entered the denervated region decreased from 58 ± 6.3 % at 30 hpf to 12.4 ± 8 % at 78 hpf (p=0.0002; Figure 2; Table S1). Most axons severed at 78 hpf initiated new growth, but actively avoided their former territories (Figure 2G–I). Avoidance was manifested as several distinct axon behaviors (Figure S2), including stalling, skirting the edge of the territory, or turning sharply, none of which were observed after axotomy at 30 hpf. Strikingly, although one-third (6/18) of axons severed at 78 hpf re-entered their former territory, reinnervation was always transient: axons that entered the denervated territory either retracted (2/6; Figure S2F–J), degenerated locally (2/6), or the parent neuron died (2/6; Figure S2K–O). Neither phagocytic blood cells nor peripheral myelin were responsible for inhibition, since avoidance was still observed in *cloche* mutant fish, which lack all blood cells [9], and in fish treated with the drug AG1478, which lack peripheral myelination (Figure S3; Table S2) [10]. Axon avoidance of former territory was a persistent effect, as axons imaged for five days after 78 hpf axotomy never reinnervated their former territory (n=7; Figure S4).

### Developmental regulation of axon plasticity and tiling

During early larval zebrafish development, trigeminal axons repel one another to partition epidermal territory [7]. When arbors are removed early in development, neighboring axons expand to fill denervated territory. This strategy for partitioning arbor territories, known as “tiling”, is often employed when multiple neurons of the same type innervate a two dimensional target. Although the arbors of some tiled neurons retain plasticity into adulthood [e.g. 11, 12], allowing them to compensate for the loss of neighbors and create new innervation patterns after injury, many populations of tiled neurites cannot reorganize after developmental critical periods [e.g. 13, 14, 15].

**Figure 3:**
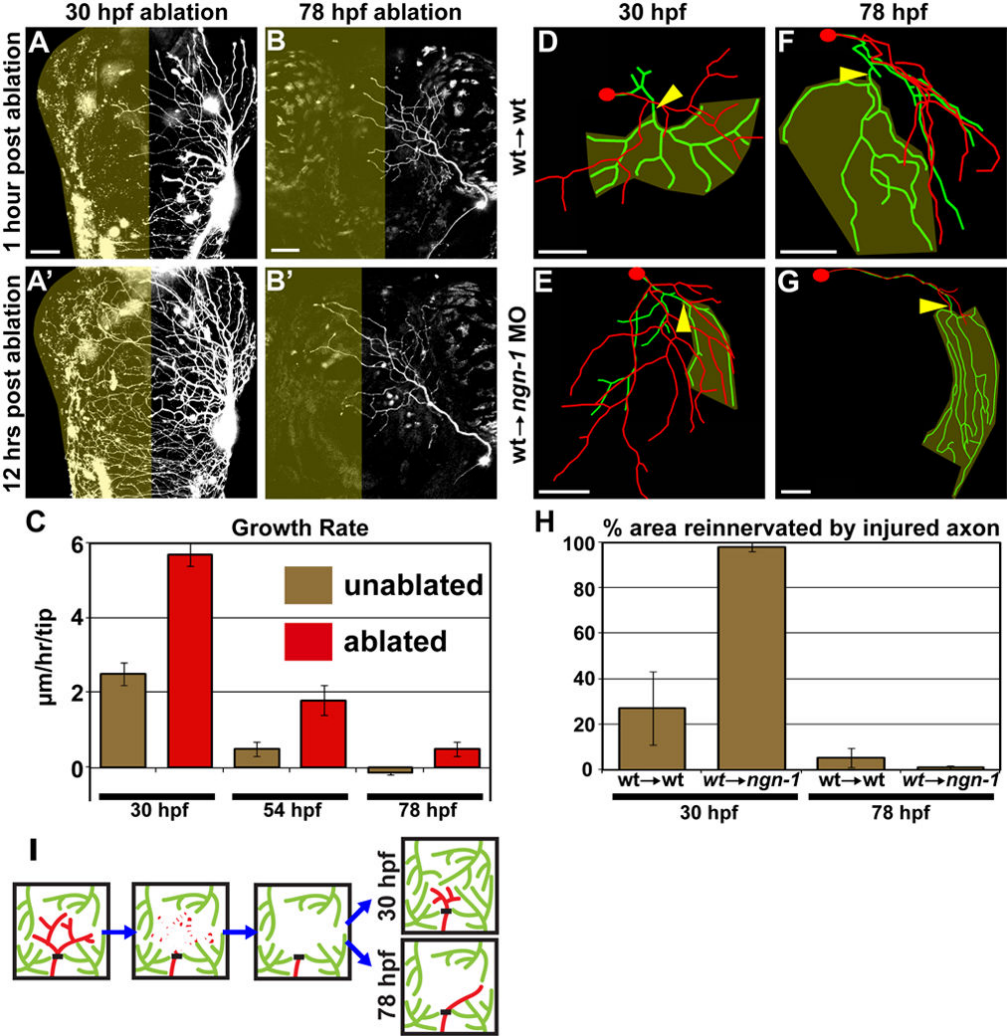
Developmental regulation of territory reinnervation strategy (A–C) Growth potential of uninjured axons is developmentally regulated. (A and B) Confocal projections. Dorsal view of zebrafish head, anterior up. Olive indicates denervated half of head. Scale bars = 50 μm. Ablation of the left trigeminal ganglion at 30 hpf (A) or 78 hpf (B). (C) Quantification of axon growth rate in unablated (olive) vs. ablated (red) animals. Values are the average growth in μm per hour of individual branch tips. Error bars ± S.E.M. See Table S3 and Movies S5–S6. (D–G) Examples of control axons (wildtype cells transplanted into wildtype host) axotomized at 30 hpf (D) or 78 hpf (F), compared to isolated regenerating axons (wildtype cells transplanted into *ngn-1* morphant host) axotomized at 30 hpf (E) or 78 hpf (G). Tracing overlays as in Figure 2. Arrowhead is site of axotomy, olive marks denervated territory, and scale bars = 50 μm. (H) Quantification of the area reinnervated by the injured axon, calculated as in Figure 2J. Error bars ± S.E.M. See Table S1 and Movies S7–S10. (I) Model of the developmental regulation of skin reinnervation by the terminal arbors of peripheral sensory axons. Black bar indicates site of axotomy. Injured axons are red, uninjured axons are green.

To characterize the territory partitioning strategy of trigeminal sensory terminals after injury at different developmental stages, we first investigated the ability of uninjured axons to sprout into newly denervated territory. To accomplish this, we laser ablated every cell within a trigeminal ganglion (Figure S1E,F) and calculated the axon growth rate from the uninjured contralateral ganglion into newly denervated territory. When a trigeminal ganglion was ablated at 30 hpf, uninjured axons from the contralateral ganglion reinnervated most of the denervated skin within 12 hours (Figure 3A). Following ablation at 78 hpf, growth into denervated territory was markedly reduced and very little branching occurred (Figure 3B–C; Table S3; μm/hr/tip: 30 hpf 5.7 ± 0.3, 78 hpf 0.5 ± 0.2; p=0.0071). Thus, there is a critical period after which the growth of uninjured axons into denervated territory diminished severely.

To test directly the hypothesis that uninjured and regenerating axons compete for denervated territory after injury at 30 hpf, we transplanted wildtype cells into *neurogenin-1* morphants, which lack somatosensory neurons, generating zebrafish with a single trigeminal neuron [7]. Strikingly, isolated axons severed at 30 hpf reinnervated virtually all (98.1 ± 1.9 %) of their former territories and grew beyond them (Figure 3E,H). In contrast, control axons (wildtype cells transplanted into wildtype embryos) only partially (27.3 ± 16.1 %; p= 0.008) reinnervated their former territories (Figure 3D,H). Thus, incomplete reinnervation of former territory at 30 hpf was not due to a non-permissive environment in the denervated region, but rather to competition with uninjured neighboring axons (Figure 3I).

The peripheral axons of isolated neurons grow until they fill the entire head, long past 78 hpf [7]. However, following axotomy of isolated arbors at 78 hpf, regenerating axons avoided their former territories, with no significant change in the percent area reinnervated compared to wildtype control transplants (1.04 ± 0.8 % vs. 5.4 ± 4.3 %; p=0.3387; Figure 3F–H). These results demonstrate that the denervated region actively repels regenerating axons at older developmental stages, and that neither diminished growth rate nor competition from uninjured neighboring axons explains the lack of reinnervation (Figure 3I). Thus, contrary to expectation, the PNS is not always a permissive environment for regeneration: At later larval stages local factors persistently marking former territories repel regenerating axons.

### A Nogo Receptor/RhoA pathway is required for inhibition of skin reinnervation

We hypothesized that axons in the CNS and PNS use similar molecular mechanisms to respond to inhibitors in their respective environments. In the CNS, myelin-associated inhibitors activate a receptor complex that includes NgR and LINGO-1 to block axon regeneration [16–19]. This complex activates an intracellular signaling cascade that involves the small GTPase RhoA, Rho kinase, and Collapsin Response Mediator Protein 2 (CRMP-2) to cause growth cone collapse [20, 21]. Zebrafish trigeminal neurons express homologs of Nogo Receptor (ZF NgR), LINGO-1 (LINGO-1a), and CRMP-2 during larval stages [22–26].

**Figure 4:**
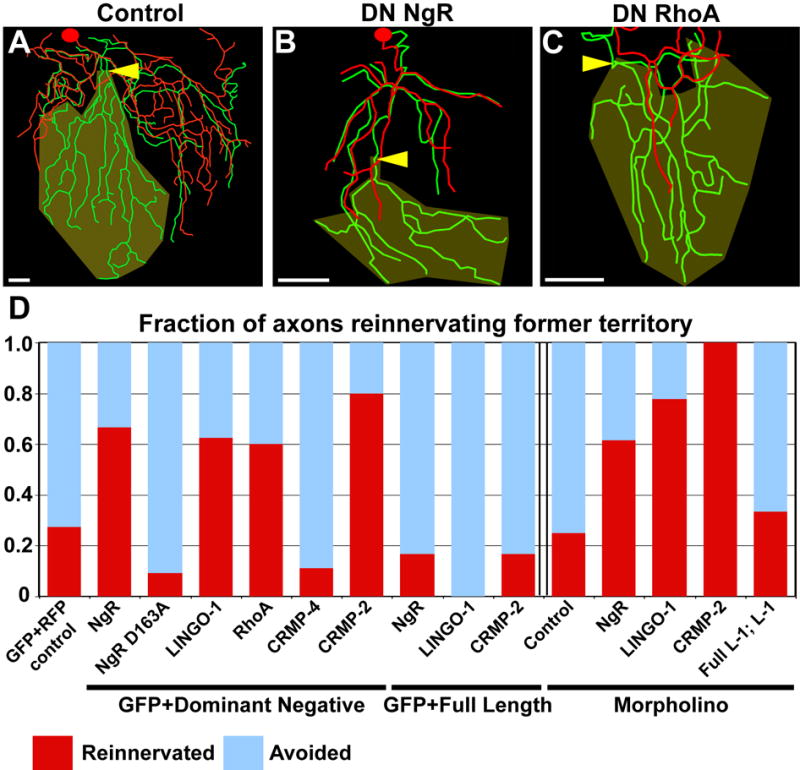
Inhibition of skin reinnervation by injured axons is mediated by the NgR/RhoA pathway (A–C) Tracing overlays as in Figure 2. Arrowhead is site of axotomy, olive marks denervated territory, and scale bars = 50 μm. 78 hpf axotomy of a trigeminal neuron expressing GFP and RFP (A), DN NgR (B), or DN RhoA (C). (D) Quantification of fraction of axons that entered the denervated territory. Data to the left of the double bar were from axons expressing GFP and dominant negative or full length versions of the genes indicated. Data to the right of the double bar were from Tg(sensory:GFP) embryos injected with indicated morpholinos. Red indicates fraction of axons that grew into denervated territory, blue indicates axons that avoided denervated territory (See supplemental methods). See Table S2 and Movies S11–S13.

To test whether the NgR pathway functions in peripheral sensory axon regeneration, we misexpressed dominant negative (DN) versions of ZF NgR, LINGO-1a and CRMP-2, as well as human RhoA, in trigeminal neurons, along with GFP [27–30]. Expression of all the DN transgenes increased the fraction of regenerating axons that entered their former territories at 78 hpf, compared to control axons co-expressing GFP and RFP, or axons expressing full length genes (Figure 4; Figure S5; Table S2). As an additional control, we mutated a conserved amino acid in the DN NgR transgene (D163A) required for NgR binding to LINGO-1 and all known NgR ligands [31]. Axons expressing DN NgR D163A avoided their former territory, similar to controls (Figure 4B,D; Figure S5D,K; Table S2) and significantly different from those expressing DN NgR. Blocking Rho kinase (ROCK) with the specific inhibitor Y-27632 [32] also improved reinnervation (Figure S6; Table S2).

To rule out non-specific effects of DN transgenes, we further investigated the function of ZF NgR, LINGO-1a, and CRMP-2 with morpholino antisense oligonucleotides (MO) targeting those genes. In all three cases, regenerating axons grew into denervated territory better than axons injected with a control MO (Figure 4; Figure S5G–J; Table S2). Expression of the full length LINGO-1a cDNA in neurons rescued the defect in repulsion observed in LINGO-1a morphants (Figure 4; Figure S5E,I,L, Table S2). These results support the idea that the NgR/RhoA pathway functions in neurons to respond to inhibitors of axon regeneration in the skin.

To determine when this pathway is required to inhibit outgrowth of trigeminal axons in the skin, we injected Y-27632 into embryos 2 hours before axotomy, as well as 1, 4, or 12 hours after axotomy (Figure S6; Table S2). Reinnervation was only improved when ROCK was inhibited ≤4 hrs after axotomy. To determine whether the same pathway was responsible for diminishing the ability of intact neighboring axons to sprout at 78 hpf, Y-27632 was injected 2 hours before ablation of an entire trigeminal ganglion. ROCK inhibition had no effect on the ability of uninjured axons from the intact ganglion to sprout across the midline (Table S3). Similarly, intact axons in LINGO-1a morphants did not grow into denervated territory after ablating a ganglion (Table S3). These results indicate that distinct pathways regulate the ability of injured and intact axon arbors to grow into denervated territory.

## Discussion

It is generally believed that, in contrast to the CNS, the periphery is permissive for axon regeneration. Although substantial functional recovery from PNS injury can occur in adulthood, it is often incomplete [reviewed in 2]. Two phenomena are known to contribute to suboptimal PNS axon regeneration: the degeneration of supportive conduits that guide axons to the periphery and the inability of regenerating sensory endings to penetrate the epidermis [1–6]. We show here that inhibitory regions of skin can also be an obstacle to peripheral reinnervation. After a developmental critical period, regenerating cutaneous sensory axon endings specifically avoid reinnervating their former territories. There is a crucial distinction between regeneration and reinnervation: severing an axon stimulates its growth, but factors in the original territory prevent reinnervation. Interestingly, severing axons in the CNS also stimulates exploratory activity, but very little reinnervation occurs [33], perhaps because axons are repelled by inhibitors expressed by myelin. When a regenerating trigeminal peripheral axon does grow into its former territory, it inevitably retracts or the cell dies, suggesting that repellents in this skin region may also be toxic to neurons. These local impediments to full reinnervation of the skin may contribute to deficits in functional recovery from peripheral injury [2].

Since inhibition is limited to a region around a degenerated axon’s original territory, we speculate that axons alter the extracellular matrix (ECM), leaving behind a persistent “ghost” that demarcates their former territories. This local mark of an axon’s territory might be laid down at a specific developmental stage to stabilize arbors. One possibility is that inhibitory factors derive from membrane-associated proteins mediating isoneuronal axon repulsion, perhaps after their ectodomains are shed from the membrane. Alternatively, axons may secrete distinct molecules into the ECM, or induce the surrounding epidermal cells to secrete inhibitors.

Perturbing Nogo receptor (NgR) signaling improved the ability of injured peripheral axons to reinnervate former territories. Several members of the Nogo Receptor (NgR) pathway, including NgR itself, its co-receptor LINGO-1, and the intracellular signaling molecules RhoA, Rho kinase and CRMP-2, are at least partially required in axons for avoidance of former territories in the skin. In the CNS this pathway is involved in responses to myelin-associated proteins that inhibit axon regeneration [21]. Thus, the central and peripheral branches of somatosensory neurons use similar mechanisms to respond to inhibitors in distinct environments. It is possible that known ligands of the NgR complex, expressed in a different context, inhibit peripheral regeneration, but because these proteins are structurally diverse, it is also possible that different ligands functions in the periphery.

Our studies reveal a critical period for the plasticity of somatosensory arbors that limits their ability to respond to injury and fully reinnervate the skin. We propose that after initial development local inhibitors stabilize sensory arbors in the skin, consequently limiting their ability to reinnervate it after injury. Several molecular studies link functional recovery and axon regeneration following spinal cord injury to developmental plasticity in the CNS [34–38]. The NgR pathway limits ocular dominance plasticity in the visual cortex [34], in addition to limiting regeneration in response to CNS injury [39, 40], and as we have now shown, PNS injury. Our results support the idea that impediments to recovery from injury to the mature PNS are a consequence of the stabilization of neuronal structure that occurs when initial morphogenesis ends.

## Experimental Procedures

A previously described GFP reporter transgene was used to visualize trigeminal neurons (Tg(sensory:GFP)) [7], as well as a variation expressing RFP (Tg(sensory:RFP)) and an Islet3-GFP line provided by Andrew Pittman and Chi-Bin Chien (Tg(Isl3:GFP))[41]. Details of time-lapse confocal imaging and 2-photon axotomy [8], data analysis, transplantation, pharmacological treatments, and morpholino and transgene design are described in supplemental data.

## Supplementary Material

01

02

03

04

05

06

07

08

09

10

11

12

13

14

## References

[1] Chen ZL, Yu WM, Strickland S (2007). Peripheral
Regeneration. Annu Rev Neurosci.

[2] Hoke A (2006). Mechanisms of disease: what
factors limit the success of peripheral nerve regeneration in
humans?. Nat Clin Pract
Neurol.

[3] Navarro X, Verdu E, Wendelschafer-Crabb G, Kennedy WR (1997). Immunohistochemical study of skin
reinnervation by regenerative axons. J Comp
Neurol.

[4] Rajan B, Polydefkis M, Hauer P, Griffin JW, McArthur JC (2003). Epidermal reinnervation after
intracutaneous axotomy in man. J Comp
Neurol.

[5] Speidel CC (1964). In vivo studies of myelinated
nerve fibers. Int Rev
Cytol.

[6] Verdu E, Navarro X (1997). Comparison of immunohistochemical
and functional reinnervation of skin and muscle after peripheral nerve
injury. Exp
Neurol.

[7] Sagasti A, Guido MR, Raible DW, Schier AF (2005). Repulsive interactions shape the
morphologies and functional arrangement of zebrafish peripheral sensory
arbors. Curr
Biol.

[8] O’Brien GS, Rieger S, Martin SM, Cavanaugh AM, Portera-Cailliau C, Sagasti A (2009). Two-photon axotomy and time-lapse
confocal imaging in live zebrafish embryos. J Vis
Exp.

[9] Stainier DY, Weinstein BM, Detrich HW, Zon LI, Fishman MC (1995). Cloche, an early acting zebrafish
gene, is required by both the endothelial and hematopoietic
lineages. Development.

[10] Lyons DA, Pogoda HM, Voas MG, Woods IG, Diamond B, Nix R, Arana N, Jacobs J, Talbot WS (2005). erbb3 and erbb2 are essential for
schwann cell migration and myelination in
zebrafish. Curr
Biol.

[11] Blackshaw SE, Nicholls JG, Parnas I (1982). Expanded receptive fields of
cutaneous mechanoreceptor cells after single neurone deletion in leech
central nervous system. J
Physiol.

[12] Scott SA, Macintyre L, Diamond J (1981). Competitive reinnervation of
salamander skin by regenerating and intact mechanosensory
nerves. Proc R Soc Lond B Biol
Sci.

[13] Eysel UT, Peichl L, Wassle H (1985). Dendritic plasticity in the early
postnatal feline retina: quantitative characteristics and sensitive
period. J Comp
Neurol.

[14] Jackson PC, Diamond J (1981). Regenerating axons reclaim
sensory targets from collateral nerve
sprouts. Science.

[15] Sugimura K, Yamamoto M, Niwa R, Satoh D, Goto S, Taniguchi M, Hayashi S, Uemura T (2003). Distinct developmental modes and
lesion-induced reactions of dendrites of two classes of Drosophila sensory
neurons. J
Neurosci.

[16] Filbin MT (2003). Myelin-associated inhibitors of
axonal regeneration in the adult mammalian CNS. Nat
Rev
Neurosci.

[17] Fournier AE, GrandPre T, Strittmatter SM (2001). Identification of a receptor
mediating Nogo-66 inhibition of axonal
regeneration. Nature.

[18] Liu BP, Fournier A, GrandPre T, Strittmatter SM (2002). Myelin-associated glycoprotein as
a functional ligand for the Nogo-66
receptor. Science.

[19] Wang KC, Koprivica V, Kim JA, Sivasankaran R, Guo Y, Neve RL, He Z (2002). Oligodendrocyte-myelin
glycoprotein is a Nogo receptor ligand that inhibits neurite
outgrowth. Nature.

[20] Mimura F, Yamagishi S, Arimura N, Fujitani M, Kubo T, Kaibuchi K, Yamashita T (2006). Myelin-associated glycoprotein
inhibits microtubule assembly by a Rho-kinase-dependent
mechanism. J Biol
Chem.

[21] Niederost B, Oertle T, Fritsche J, McKinney RA, Bandtlow CE (2002). Nogo-A and myelin-associated
glycoprotein mediate neurite growth inhibition by antagonistic regulation of
RhoA and Rac1. J
Neurosci.

[22] Brosamle C, Halpern ME (2008). Nogo-Nogo receptor signalling in
PNS axon outgrowth and pathfinding. Mol Cell
Neurosci.

[23] Christie TL, Starovic-Subota O, Childs S (2006). Zebrafish collapsin response
mediator protein (CRMP)-2 is expressed in developing
neurons. Gene Expr
Patterns.

[24] Klinger M, Taylor JS, Oertle T, Schwab ME, Stuermer CA, Diekmann H (2004). Identification of Nogo-66
receptor (NgR) and homologous genes in fish. Mol Biol
Evol.

[25] Schweitzer J, Becker CG, Schachner M, Becker T (2005). Expression of collapsin response
mediator proteins in the nervous system of embryonic
zebrafish. Gene Expr
Patterns.

[26] Thisse B, Wright GJ, Thisse C (2008). Embryonic and Larval Expression
Patterns from a Large Scale Screening for Novel Low Affinity Extracellular
Protein Interactions. ZFIN Direct Data
Submission.

[27] Domeniconi M, Cao Z, Spencer T, Sivasankaran R, Wang K, Nikulina E, Kimura N, Cai H, Deng K, Gao Y, He Z, Filbin M (2002). Myelin-associated glycoprotein
interacts with the Nogo66 receptor to inhibit neurite
outgrowth. Neuron.

[28] Inagaki N, Chihara K, Arimura N, Menager C, Kawano Y, Matsuo N, Nishimura T, Amano M, Kaibuchi K (2001). CRMP-2 induces axons in cultured
hippocampal neurons. Nat
Neurosci.

[29] Mi S, Lee X, Shao Z, Thill G, Ji B, Relton J, Levesque M, Allaire N, Perrin S, Sands B, Crowell T, Cate RL, McCoy JM, Pepinsky RB (2004). LINGO-1 is a component of the
Nogo-66 receptor/p75 signaling complex. Nat
Neurosci.

[30] Qiu RG, Chen J, McCormick F, Symons M (1995). A role for Rho in Ras
transformation. Proc Natl Acad Sci U S
A.

[31] Lauren J, Hu F, Chin J, Liao J, Airaksinen MS, Strittmatter SM (2007). Characterization of myelin ligand
complexes with neuronal Nogo-66 receptor family
members. J Biol
Chem.

[32] Uehata M, Ishizaki T, Satoh H, Ono T, Kawahara T, Morishita T, Tamakawa H, Yamagami K, Inui J, Maekawa M, Narumiya S (1997). Calcium sensitization of smooth
muscle mediated by a Rho-associated protein kinase in
hypertension. Nature.

[33] Kerschensteiner M, Schwab ME, Lichtman JW, Misgeld T (2005). In vivo imaging of axonal
degeneration and regeneration in the injured spinal
cord. Nat
Med.

[34] McGee AW, Yang Y, Fischer QS, Daw NW, Strittmatter SM (2005). Experience-driven plasticity of
visual cortex limited by myelin and Nogo
receptor. Science.

[35] Pizzorusso T, Medini P, Berardi N, Chierzi S, Fawcett JW, Maffei L (2002). Reactivation of ocular dominance
plasticity in the adult visual
cortex. Science.

[36] Atwal JK, Pinkston-Gosse J, Syken J, Stawicki S, Wu Y, Shatz C, Tessier-Lavigne M (2008). PirB is a functional receptor for
myelin inhibitors of axonal
regeneration. Science.

[37] Syken J, Grandpre T, Kanold PO, Shatz CJ (2006). PirB restricts ocular-dominance
plasticity in visual
cortex. Science.

[38] Bradbury EJ, Moon LD, Popat RJ, King VR, Bennett GS, Patel PN, Fawcett JW, McMahon SB (2002). Chondroitinase ABC promotes
functional recovery after spinal cord
injury. Nature.

[39] Cafferty WB, Strittmatter SM (2006). The Nogo-Nogo receptor pathway
limits a spectrum of adult CNS axonal growth. J
Neurosci.

[40] Kim JE, Liu BP, Park JH, Strittmatter SM (2004). Nogo-66 receptor prevents
raphespinal and rubrospinal axon regeneration and limits functional recovery
from spinal cord
injury. Neuron.

[41] Pittman AJ, Law MY, Chien CB (2008). Pathfinding in a large vertebrate
axon tract: isotypic interactions guide retinotectal axons at multiple
choice
points. Development.

