# Supplementary material for: Developmentally regulated local inhibition and loss of plasticity hinder reinnervation of the skin by injured peripheral sensory axons
Source: Curr Biol. Author manuscript; Available in PMC 2010 Dec 29. (PMC2805760; doi:10.1016/j.cub.2009.10.051)
Supplement: 01 [file NIHMS157335-supplement-01.doc]

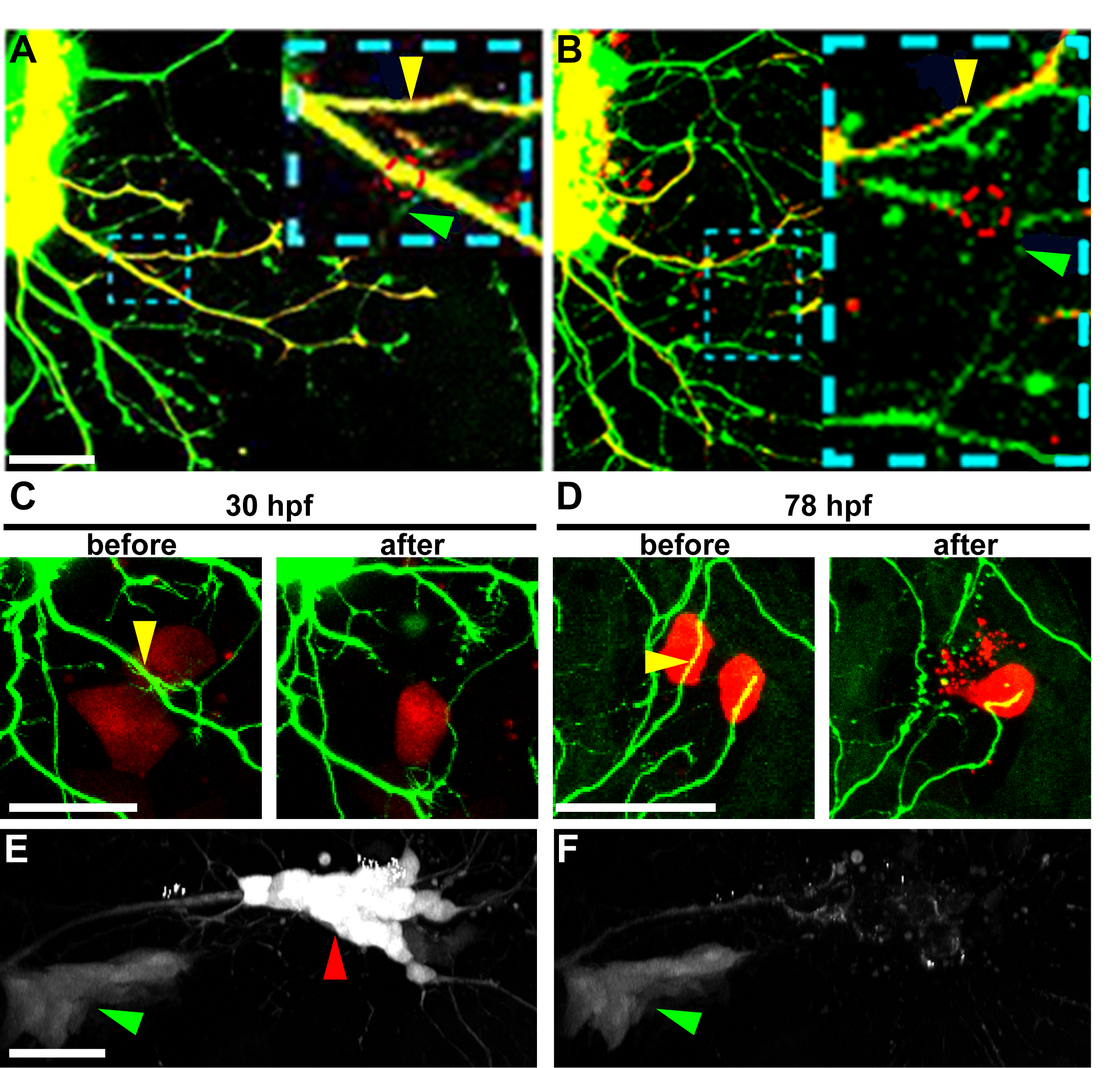


**Figure S1: Two-photon excitation precisely severs axons and causes limited tissue damage.**

(A-B) Confocal images taken before (A) and 1 hour after (B) two-photon axotomy at 30 hpf. All trigeminal neurons were labeled with GFP, and a variegated population labeled with RFP. Dashed blue box indicates region magnified in inset. Dashed red circle indicates site of axotomy. Arrowheads point to a branch of the injured axon (yellow) and a branch of a neighboring uninjured axon (green) that remained intact even though they were in close proximity (< 8 µm away) to the site of axotomy (axotomized branch in B has lost red fluorescence due to bleaching). In more than one hundred axotomies, we never observed damage to axon branches that were not specifically targeted. (C,D) Confocal images taken before and 30 minutes after 30 hpf (C) or 78 hpf (D) two-photon axotomy. Variegated population of trigeminal neurons labeled with GFP, keratinocytes labeled with mCherry. Yellow arrow indicates site of axotomy. A single skin cell directly above or below the site of axotomy died after both 30 and 78 hpf axotomy (7/7 axotomies at 30 hpf and 6/6 axotomies at 78 hpf). (E,F) Two-photon images taken before (E) and 1 hour after (F) two-photon ablation of an entire trigeminal ganglion at 30 hpf. All trigeminal neurons as well as other cranial ganglia were labeled with GFP. Red arrowhead indicates ablated ganglion. Green arrowhead indicates a neighboring cranial ganglion that remained intact. Scale bar represents 50 microns in all panels.


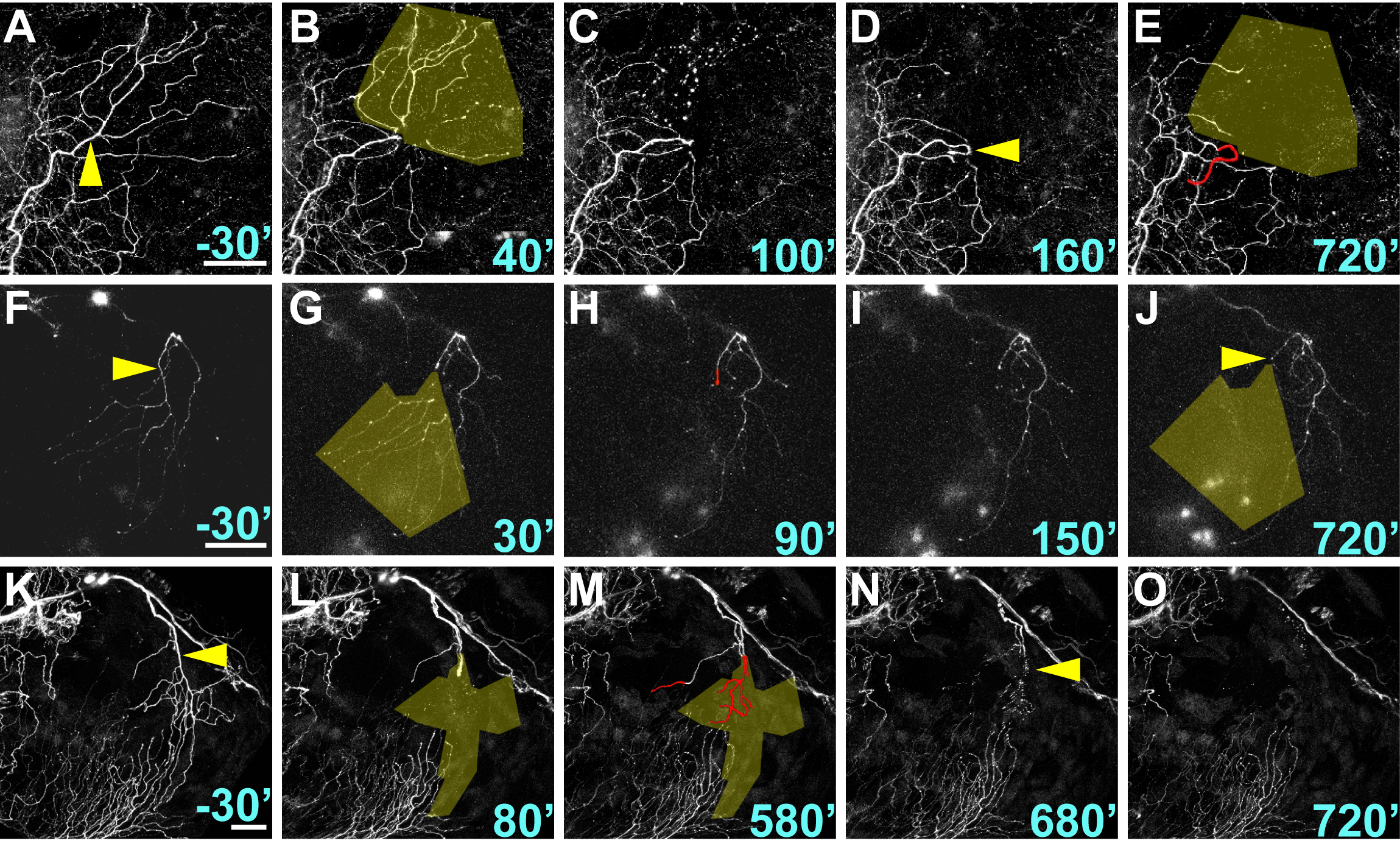


**Figure S2: Time-lapse imaging reveals diverse behaviors of regenerating axons after 78 hpf axotomy.**

Time series of confocal projections, minutes relative to axotomy displayed in lower right. Panels A, F, and K show axons before axotomy, with arrowheads pointing to site of axotomy. Olive overlay highlights denervated region. In panels E, H, and M, red indicates post-axotomy growth of injured axons. (A-E) Arrowhead in D indicates hairpin turn of regenerating axon away from the denervated region. (F-J) Regenerating axon enters the denervated territory (H) and retracts back out, ultimately stalling out at the boundary of the denervated territory (arrowhead in J). (K-O) The regenerating axon partially reinnervated the denervated region by 580 minutes after axotomy (M), followed by death of the neuron (N-O). Arrowhead in N indicates the beginning of apoptotic degeneration, characterized by distal to proximal progression of axon degeneration. By 720 minutes after axotomy, the injured axon died, leaving a denervated region of skin. Scale bars represent 50 microns.


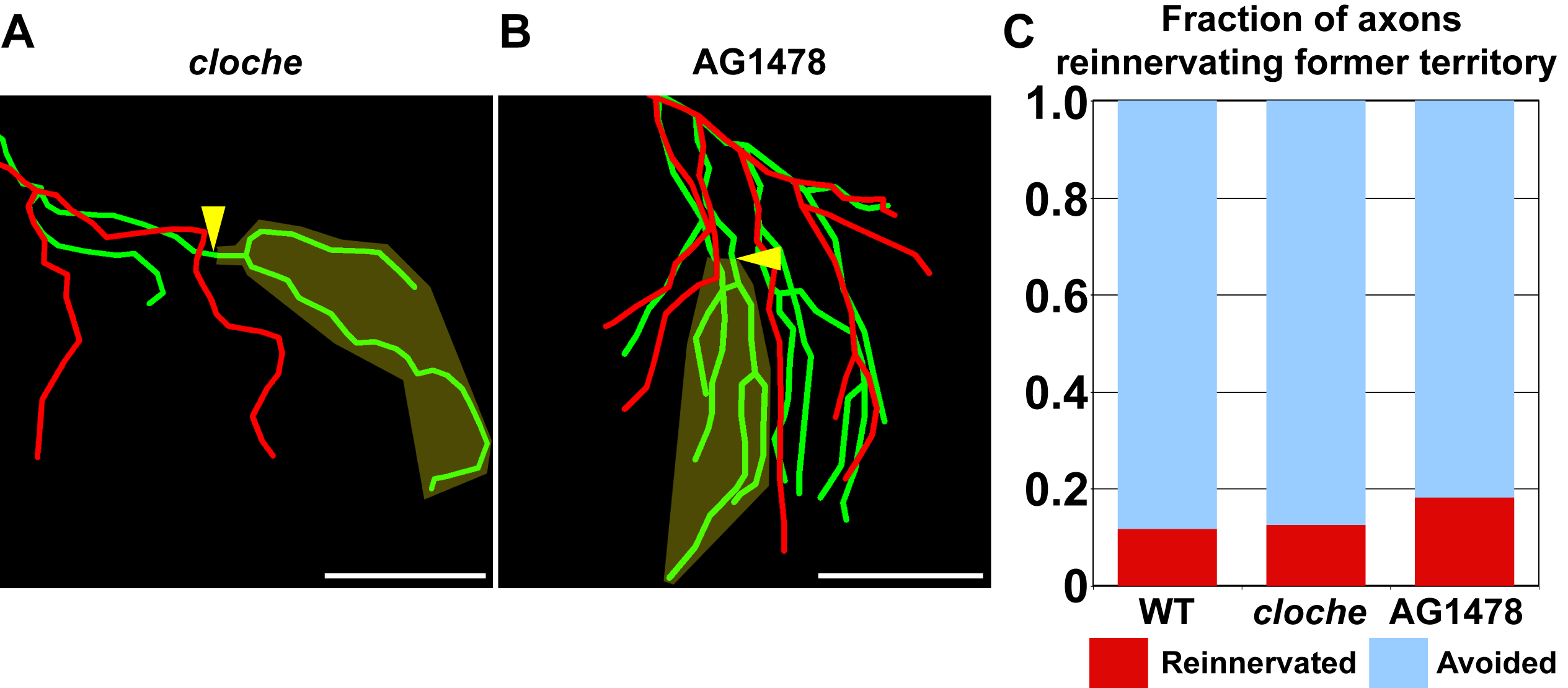


**Figure S3: Neither macrophages nor myelin are required for inhibiting axon growth into denervated territory.**

(A,B) Tracing overlay showing 3-D reconstructions of axons before axotomy (green) and 12 hours after axotomy (red), aligned at the closest shared branchpoint proximal to the site of axotomy. Arrowhead indicates site of axotomy, olive overlay marks the denervated territory, scale bar = 50 µm. All axotomies were performed at 78 hpf. A regenerating axon avoids its former territory in *cloche* mutants (A), which lack all blood cells, and in embryos treated with AG1478, which lack myelination by peripheral glia (B). (C) Quantification of fraction of axons that entered the denervated territory. Neither manipulation significantly increased the fraction of axons reinnervating former territory after 78 hpf axotomy (same wildtype data as in Figure 2): p=0.7593 (cloche), p=0.8693 (AG1478). Data included in Table S2.

**Supplemental figure 4**


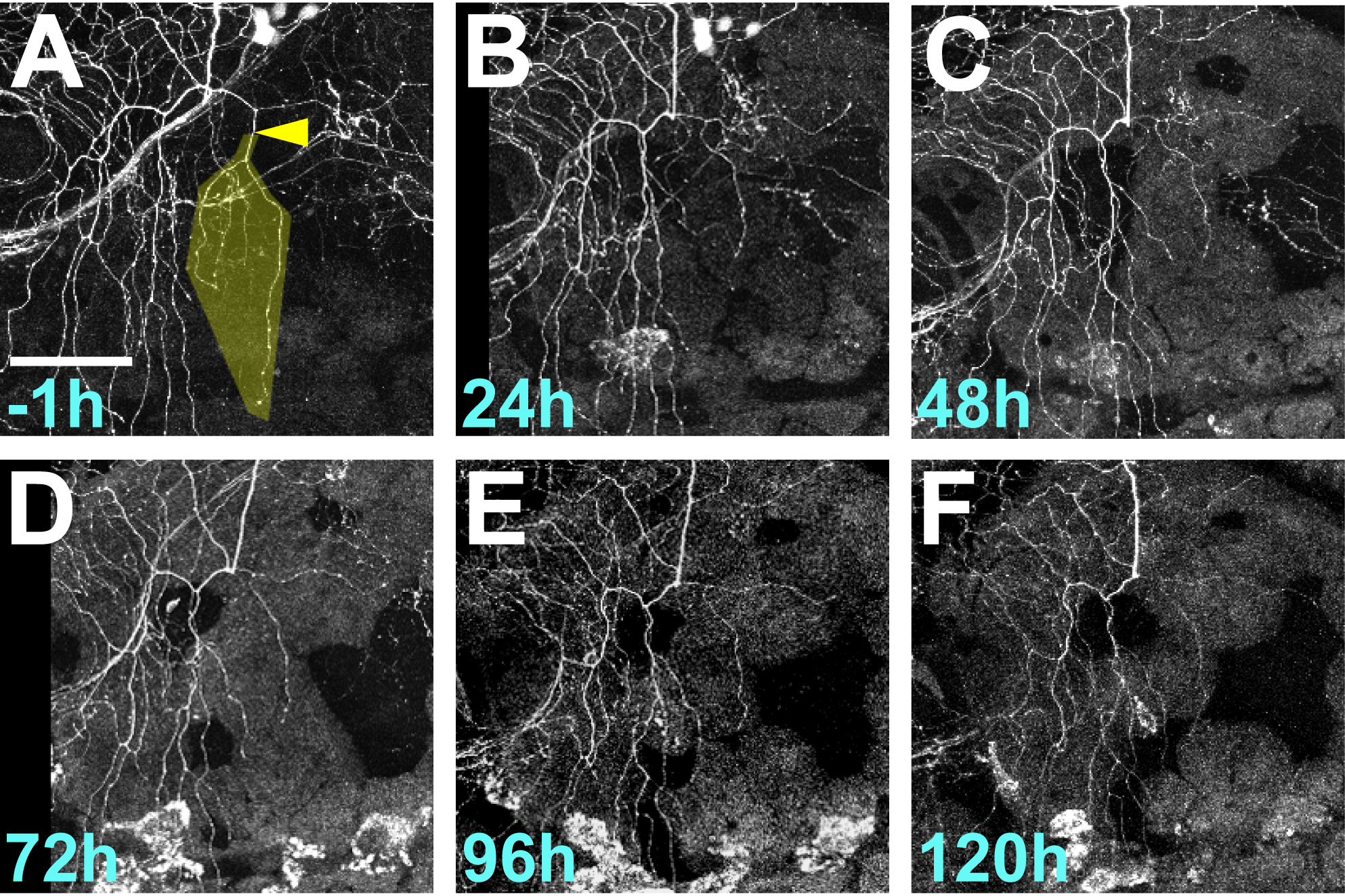


**Figure S4: Severed axons persistently fail to innervate denervated territory.**

An axon severed at 78 hpf does not appreciably grow into former territory, even after five days. Time series of confocal image stacks. Time stamps are in hours relative to axotomy at 78 hpf. Olive shading highlights the denervated region. Yellow arrowhead indicates site of axotomy. Scale bar = 50 µm.

**
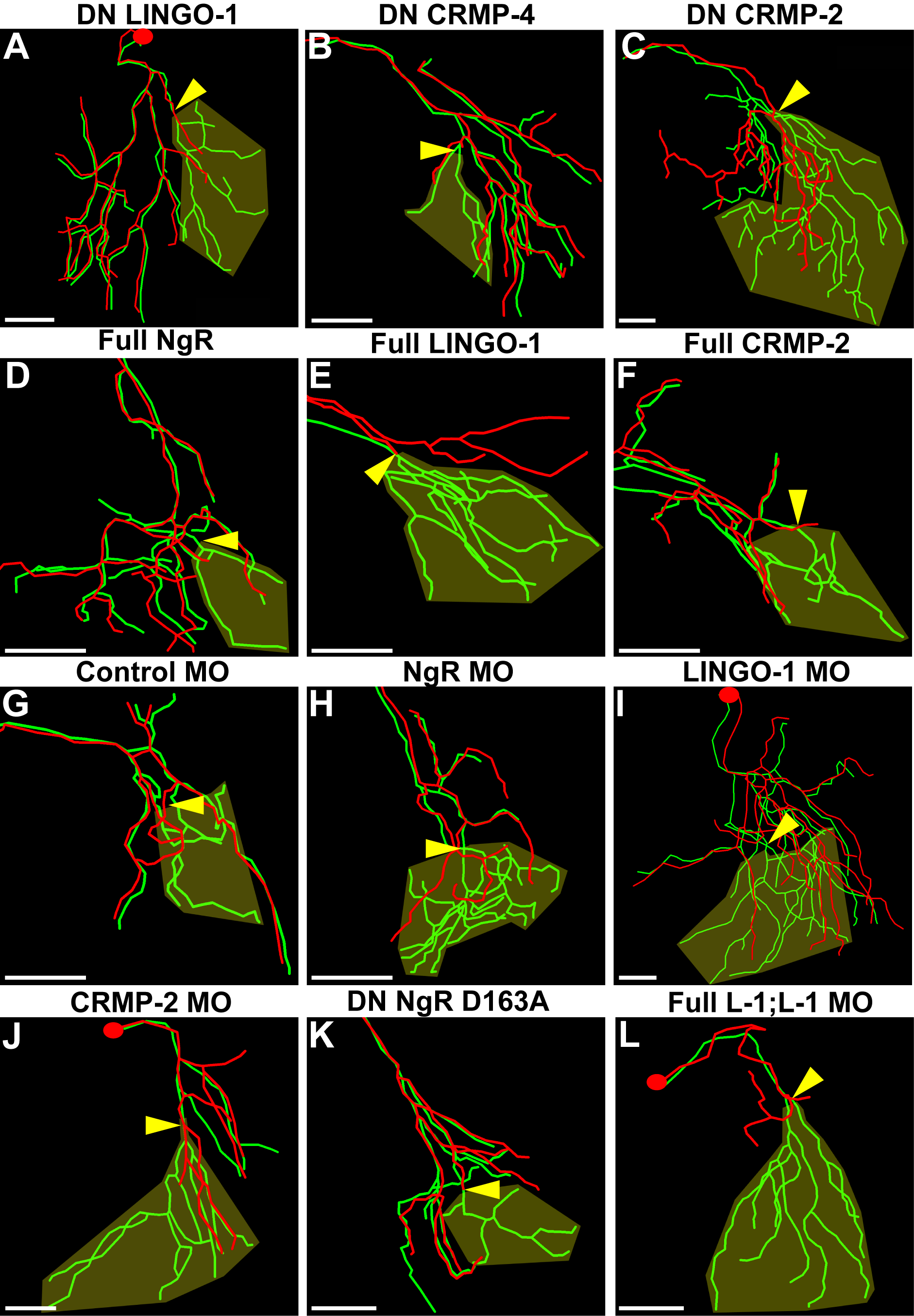
**

**Figure S5: Disrupting NgR/RhoA signaling improves reinnervation.**

Tracing overlays showing 3-D reconstructions of axons before axotomy (green) and 12 hours after axotomy (red), aligned at the shared branchpoint most proximal to the site of axotomy. Arrowhead indicates site of axotomy, olive overlay marks the denervated territory, and scale bars = 50 µm. All axotomies performed at 78 hpf. (A-F) Axotomy of a trigeminal neuron expressing GFP and DN (dominant negative) LINGO-1 (A), DN CRMP-4 (B), DN CRMP-2 (C), full length (Full) NgR (D), Full LINGO-1 (E), or Full CRMP-2 (F). (G-J) Axotomy of GFP labeled trigeminal neuron in embryos injected with a control morpholino (G), or morpholinos targeting NgR (H) LINGO-1 (I), or CRMP-2 (J). (K) Axotomy of a trigeminal neuron expressing GFP and DN NgR with a mutation (D163A) that disrupts binding to ligands and coreceptors. (L) Axotomy of a trigeminal neuron expressing GFP and DN Full LINGO-1 in an embryo injected with a morpholino targeting LINGO-1. Data included in Table S2.

**
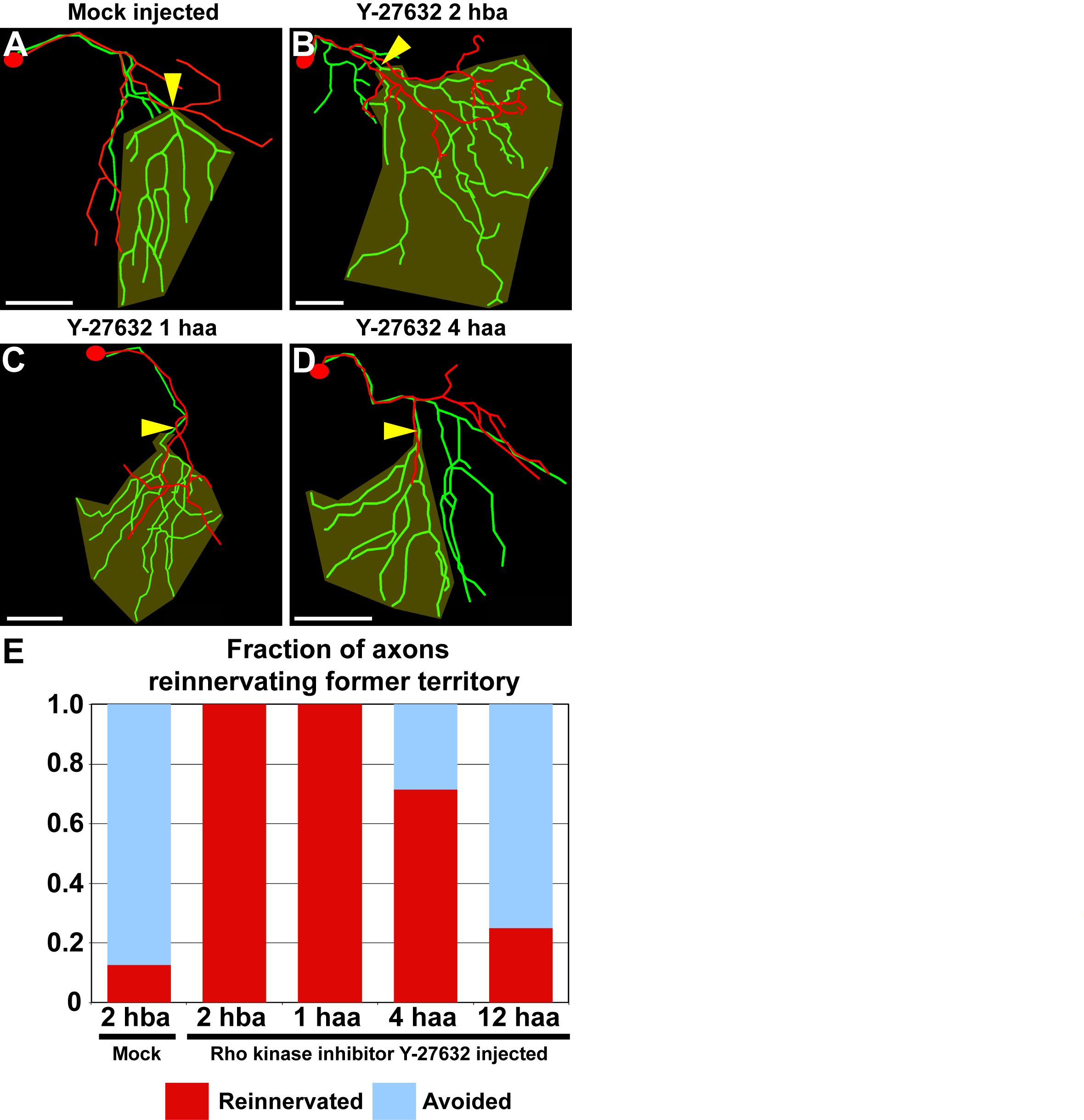
**

**Figure S6: The ROCK inhibitor Y-27632 improves reinnervation of the skin by injured axons during a specific time window.**

(A-D) Tracing overlay showing 3-D reconstructions of axons before axotomy (green) and 12 hours after axotomy (red), aligned at the shared branchpoint most proximal to the site of axotomy. Arrowhead indicates site of axotomy, olive overlay marks the denervated territory, and scale bars = 50 µm. (A) The injured axon did not reinnervate its former territory after mock injection with the vehicle (10% phenol red in water). The injured axon reinnervated a portion of its former territory when ROCK inhibitor was injected 2 hours before axotomy (hba, B), 1 hour after axotomy (haa, C), or 4 haa (D). (E) Quantification of the fraction of axons that grew into denervated territory. Data is included in Table S2.

| **Category** | **N** | **AVG % new growth in denervated territory ± S.E.M.** | **AVG % area reinnervated ± S.E.M.** |
| --- | --- | --- | --- |
| Wildtype (WT) | | | |
| 30 hpf WT | 16 | 58 ± 6.3 | 47.5 ± 7.9 |
| 54 hpf WT | 15 | 33.1 ± 9.8 | 10.5 ± 3.5 |
| 78 hpf WT | 18 | 12.4 ± 8.0 | 0.3 ± 0.2 |
| 78 hpf WT un-anesthetized | 12 | 25 ± 13.1 | 2.3 ± 1.4 |
| Transplants to generate isolated axons | | | |
| 30 hpf transplant into WT | 6 | NA | 27.3 ± 16.1 |
| 30 hpf transplant into *ngn-1* MO | 4 | NA | 98.1 ± 1.9 |
| 78 hpf transplant into WT | 5 | NA | 5.4 ± 4.3 |
| 78 hpf transplant into *ngn-1* MO | 7 | NA | 1.04 ± 0.8 |

**Table S1: Quantification of skin reinnervation after axotomy at different stages of development.**

Wildtype trigeminal peripheral arbors were severed at a ≥ 2^nd^ order branch at 30, 54, or 78 hpf. The average new axon growth that entered its former territory was calculated as a measure of target permissiveness/repulsion. The average area reinnervated was calculated as a measure of the extent of reinnervation. To rule out any effect of anesthesia used in the making of time-lapse movies, we also performed axotomies in 12 fish that were only anesthetized with tricaine during initial imaging and axotomy (about one hour), and the end-point image, twelve hours after axotomy. In the intervening time animals were removed from agarose and placed into tricaine free Ringers solution. The 78 hpf WT un-anesthetized fish did not differ in their ability to regenerate or reinnervate the skin compared to 78 hpf WT (p=0.9564).

Isolated trigeminal axons were severed at either 30 or 78 hpf. Since axons were unbound by repulsion from neighbors they grew in all directions both during development and after injury, so the % new growth in denervated territory was not an appropriate calculation in this case.

Axon arbors were quantitatively traced in three dimensions from confocal stacks using NeuroLucida software. Tracings from confocal images taken before and 12 hours after axotomy were overlayed and aligned at the shared branchpoint closest to the site of axotomy to determine how much of the regenerating axon entered denervated territory. The denervated region was determined by watching the corresponding time-lapse confocal movie, which showed the site of axotomy and the portion of the severed axon that degenerates.

To calculate the % regenerative growth that entered the denervated territory, we divided the length of new growth that entered the denervated region by the total length of new growth (both from 12 hours after axotomy confocal image). To calculate the area reinnervated by new growth, we divided the area (convex hull) of new growth (from 12 hours after axotomy tracing) by the area of the denervated region (from before axotomy tracing). Student’s t-test was used to test for significant differences between groups.

| **Category** | **N** | **Fraction of axons reinnervating former territory** | **P value** |
| --- | --- | --- | --- |
| Requirement for macrophages and glia (p value vs. wildtype) | | | |
| Wildtype (same as Table S1) | 17 | 0.12 | -- |
| *Cloche* | 11 | 0.13 | 0.7593 |
| AG1478 treated | 8 | 0.18 | 0.8693 |
| Cell autonomous misexpression (p value vs. GFP+RFP control) | | | |
| GFP+RFP control | 11 | 0.27 | -- |
| GFP+DN NgR | 6 | 0.67 | 0.0509 |
| GFP+DN LINGO-1 | 8 | 0.63 | 0.0393 |
| GFP+DN RhoA | 5 | 0.60 | 0.1289 |
| GFP+DN CRMP-4 | 9 | 0.11 | 0.9431 |
| GFP+DN CRMP-2 | 5 | 0.80 | 0.0216 |
| GFP+Full NgR | 6 | 0.17 | 0.8520 |
| GFP+Full LINGO-1 | 6 | 0.00 | 1.0000 |
| GFP+Full CRMP-2 | 6 | 0.17 | 0.8520 |
| GFP+DN NgR D163A | 11 | 0.09 | 0.0001  (vs. DN NgR) |
| Loss of function (p value vs. control MO) | | | |
| Control MO | 8 | 0.25 | -- |
| NgR MO | 13 | 0.62 | 0.0056 |
| LINGO-1 MO | 9 | 0.78 | 0.0013 |
| CRMP-2 MO | 7 | 1.00 | 0.0001 |
| GFP+Full LINGO-1;LINGO-1 MO | 9 | 0.33 | 0.0054  (vs. LINGO-1 MO) |
| Temporal requirement for Rho kinase (p value vs. mock injected control) | | | |
| Mock injected control | 8 | 0.13 | -- |
| Y-27632, 2 hrs before axotomy | 8 | 1.00 | <0.0001 |
| Y-27632, 1 hr after axotomy | 6 | 1.00 | <0.0001 |
| Y-27632, 4 hrs after axotomy | 7 | 0.71 | 0.0005 |
| Y-27632, 12 hrs after axotomy | 4 | 0.25 | 0.4138 |

**Table S2: Frequency of successful skin reinnervation after axotomy at 78 hpf.** Following axotomy at 78 hpf, there was a largely bimodal distribution of the percent of new axon growth that enters denervated territory, so binomial distribution was used to assess the significance of differences between groups (p values shown). Axons typically either completely avoided (0% growth into denervated skin) or completely entered (100%) their former territory, since axon stumps are usually oriented towards former territory. The percent of new growth that entered denervated territory was determined as in Table S1. The fraction of axons reinnervating former territory is the fraction of axons in that group in which ≥25% of the regenerated axon length was in the denervated region.

| **Category** | **# of animals** | **# of tips** | **AVG growth rate (µm/hr/tip) ± S.E.M.** |
| --- | --- | --- | --- |
| Wildtype | | | |
| 30 hpf WT | 6 | 78 | 2.5 ± 0.3 |
| 54 hpf WT | 5 | 58 | 0.5 ± 0.2 |
| 78 hpf WT | 5 | 53 | -0.14 ± 0.05 |
| Contralateral ganglion ablated | | | |
| 30 hpf ablated | 6 | 124 | 5.7 ± 0.3 |
| 54 hpf ablated | 5 | 86 | 1.8 ± 0.4 |
| 78 hpf ablated | 6 | 56 | 0.5 ± 0.2 |
| 78 hpf ablated; Y-27632 | 4 | 36 | 0.4 ± 0.5 |
| 78 hpf ablated; LINGO-1 MO | 5 | 39 | 0.6 ± 0.2 |

**Table S3: Growth rate of uninjured axons into denervated territory.**

Axon arbors were traced in three dimensions from confocal stacks collected before and 12 hours after ablation of one trigeminal ganglion. Traces were overlayed to define new axon growth, and growth rate was calculated for individual branch tips: sum of changes in length of each branch tip (length at 12 hours after ablation – length before ablation)/12 hrs/# axon tips. P values were calculated with Student’s t-test.

**Supplemental Experimental Procedures**

**Mounting zebrafish for axotomy and long term imaging:**

Zebrafish were dechorionated and anesthetized in 0.02% tricaine (Sigma, St. Louis, MO) before mounting in 1% low melting point agarose (Sigma, St. Louis, MO) in a sealed chamber. Details of the mounting and imaging technique are described elsewhere [1].

**Two-photon axotomies and cell ablations:**

Two-photon axotomy and cell ablations were performed using a custom-built two-photon microscope with a femtosecond Ti:Sapphire laser (Chameleon Ultra II, Coherent) tuned to 910 nm, a 40X water-immersion objective (0.8 NA, Olympus) and ScanImage software [2]. GFP-expressing zebrafish trigeminal neurons were identified using ~30 mW power (at the sample). Axotomy was accomplished with a single sweep at 70x ScanImage zoom with a power of ~180 mW. Cell ablations were performed with a single sweep at 20x ScanImage zoom. To ablate an entire ganglion, this process was repeated until all 30 cell bodies in the ganglia were ablated. Details of the axotomy and ablation procedure are reported elsewhere [1].

**Time-lapse confocal imaging:**

Embryos were imaged every 20-30 minutes for at least 12 hours using a 20X air objective (Zeiss, Thornwood, NY). A heated stage maintained fish at ~28 ^o^C. Approximately 60-80 confocal sections (3.5 µm apart) were collected at each time point using a Zeiss LSM 510 confocal microscope with Multi Time software (Thornwood, NY). Maximum intensity projections of confocal stacks were compiled into movies using ImageJ software (http://rsb.info.nih.gov/ij/).

**Axon tracing and data analysis:**

NeuroLucida software (Microbrightfield, Williston, VT) was used to generate tracings of trigeminal neurons from confocal stacks. Length and convex hull measurements from tracings were used to quantify growth of regenerating and uninjured neurons, the percentage of the regenerating axon that entered denervated territory, and the area of skin reinnervated by the injured axon (see figure legend 2J,K). To minimize distortion caused by growth of the head, pre- and 12 hrs post- axotomy pictures were aligned at the closest shared branchpoint proximal to the site of axotomy. Significance was set at p<0.05. Error bars in figures are standard error of the mean.

**Transplants to generate zebrafish with a single trigeminal neuron:**

Wildtype cells from a sensory-GFP transgenic embryo were transplanted into embryos (~1000 cell stage) injected with 16 ng of a morpholino that blocks translation of the *neurogenin-1* (*ngn-1*) gene (Gene Tools, Philomath, OR), which is required for the development of all peripheral sensory neurons [3, 4]. Transplant recipients were screened with a fluorescence dissecting scope (Zeiss, Thornwood, NY) for the presence of a single GFP-labeled trigeminal neuron and subjected to axotomy at 30 or 78 hpf.

**Pharmacological treatments:**

250 μM Y-27632 (Sigma, St. Louis, MO) was injected directly into the zebrafish heart at defined times before or after axotomy. Zebrafish were dechorionated and anaesthetized in 0.02% tricaine, mounted in slanted agarose wells ventral side up so that the heart could be clearly visualized through a dissecting microscope. 10 nL of the drug in 10% phenol red was pressure injected with a single pulse from a microinjector into the zebrafish heart. Embryos were bathed continuously in 5 uM AG1478 (Sigma, St. Louis, MO) starting at 10 hpf. Myelin basic protein staining was used to verify the absence of peripheral myelin [5].

**Dominant negative transgenes and full-length cDNAs:**

Dominant negative NgR, LINGO-1, CRMP-4, and CRMP-2 transgenes were made from regions of zebrafish cDNAs homologous to regions of mammalian genes previously shown to function as dominant negatives [6-9]. PCR was used to amplify these regions from zebrafish cDNA. Xho1 sites were appended to primers for subsequent subcloning. The following primer sequences were used for amplification (Operon, Huntsville, AL): NgR 5’ end ATGGAGACCTTAATCGTGGAG, NgR 3’end TCAACAACCTTCCAAATCATCGCT; LINGO-1 5’ end ACCATGGTGGCAGGGGAAGT, LINGO-1 3’ end TCAGCCCGTCTTTATATCGAATGG; CRMP-4 5’ end CCATGTCTTACCAAGGCAAA, CRMP-4 3’ end TCACCATTTACTCCAGCGATCGA; CRMP-2 5’ end CGAAGATGTCTGGCTATCAGG, CRMP-2 3’end TCAGGTGAAGTCATCTTTACCAAC. Full length cDNAs were amplified using the following primers for the 3’ end: Full NgR CTAGGACAAAGCCAAAGACA; Full LINGO-1 TTATATCATTTTCATGTTAAA; Full CRMP-2 TTTAGCCCAGGCTGGTGA.

Amplified cDNA was subcloned into a vector downstream of 14 copies of the Gal4 Upstream Activating Sequence, along with GFP. In a separate transgene, a sensory neuron specific promoter was used to drive the GAL4-VP16 activator. Co-injection of these transgenes at the one-cell stage resulted in transient mosaic expression in embryonic and larval somatosensory neurons.

Site directed mutagenesis of the DN NgR transgene was done using the New England Biolabs kit (Ipswich, MA). A conserved residue (163 in human and zebrafish proteins) was mutagenized from aspartic acid (D) to alanine (A) [10]. Mutagenesis primer ATCTGTACCTGCAGGCCAACAACCTAC; reverse primer ACTGAAGTGAAAAGAGTCCTCG.

**Morpholino Design:**

The NgR and LINGO-1 ATG morpholinos were directed to the start site; CTCCACGATTAAGGTCTTCATGTTT [11] and CACTCACTTCCCCTGCCACCATCCT, respectively. The CRMP-2 splice morpholino was directed to the intron 1-2/exon 2 boundary; CACTCTGGAAACACAGATAAACACA. The control morpholino differed from the CRMP-2 morpholino by five base pairs; CAGTCTCGAAACAGAGAAAAAGACA (Gene Tools, Philomath, OR). For each morpholino, 4-8 ng were injected at the single cell stage.

**Supplemental References**

1. O'Brien, G.S., Rieger, S., Martin, S.M., Cavanaugh, A.M., Portera-Cailliau, C., and Sagasti, A. (2009). Two-photon axotomy and time-lapse confocal imaging in live zebrafish embryos. J Vis Exp, doi: 10.3791/1129.

2. Pologruto, T.A., Sabatini, B.L., and Svoboda, K. (2003). ScanImage: flexible software for operating laser scanning microscopes. Biomed Eng Online *2*, 13.

3. Andermann, P., Ungos, J., and Raible, D.W. (2002). Neurogenin1 defines zebrafish cranial sensory ganglia precursors. Dev Biol *251*, 45-58.

4. Cornell, R.A., and Eisen, J.S. (2002). Delta/Notch signaling promotes formation of zebrafish neural crest by repressing Neurogenin 1 function. Development *129*, 2639-2648.

5. Lyons, D.A., Pogoda, H.M., Voas, M.G., Woods, I.G., Diamond, B., Nix, R., Arana, N., Jacobs, J., and Talbot, W.S. (2005). erbb3 and erbb2 are essential for schwann cell migration and myelination in zebrafish. Curr Biol *15*, 513-524.

6. Alabed, Y.Z., Pool, M., Ong Tone, S., and Fournier, A.E. (2007). Identification of CRMP4 as a convergent regulator of axon outgrowth inhibition. J Neurosci *27*, 1702-1711.

7. Domeniconi, M., Cao, Z., Spencer, T., Sivasankaran, R., Wang, K., Nikulina, E., Kimura, N., Cai, H., Deng, K., Gao, Y., He, Z., and Filbin, M. (2002). Myelin-associated glycoprotein interacts with the Nogo66 receptor to inhibit neurite outgrowth. Neuron *35*, 283-290.

8. Inagaki, N., Chihara, K., Arimura, N., Menager, C., Kawano, Y., Matsuo, N., Nishimura, T., Amano, M., and Kaibuchi, K. (2001). CRMP-2 induces axons in cultured hippocampal neurons. Nat Neurosci *4*, 781-782.

9. Mi, S., Lee, X., Shao, Z., Thill, G., Ji, B., Relton, J., Levesque, M., Allaire, N., Perrin, S., Sands, B., Crowell, T., Cate, R.L., McCoy, J.M., and Pepinsky, R.B. (2004). LINGO-1 is a component of the Nogo-66 receptor/p75 signaling complex. Nat Neurosci *7*, 221-228.

10. Lauren, J., Hu, F., Chin, J., Liao, J., Airaksinen, M.S., and Strittmatter, S.M. (2007). Characterization of myelin ligand complexes with neuronal Nogo-66 receptor family members. J Biol Chem *282*, 5715-5725.

11. Brosamle, C., and Halpern, M.E. (2008). Nogo-Nogo receptor signalling in PNS axon outgrowth and pathfinding. Mol Cell Neurosci.
